# Family physicians perceived role in perinatal mental health: an integrative review

**DOI:** 10.1186/s12875-018-0843-1

**Published:** 2018-09-08

**Authors:** Maria Noonan, Owen Doody, Julie Jomeen, Andrew O’Regan, Rose Galvin

**Affiliations:** 1 Department of Nursing and Midwifery, Faculty of Education & Health Sciences Health Sciences Building, University of Limerick, Limerick, Ireland; 2 Faculty of Health and Social Care, University of Hull, Hull, UK; 3 Graduate Entry Medical School, Faculty of Education & Health Sciences, University of Limerick, Limerick, Ireland; 4 School of Allied Health, Faculty of Education & Health Sciences, Health Sciences Building, University of Limerick, Limerick, Ireland

**Keywords:** Integrative review, Family physician, General practitioner, Perinatal mental health, Postpartum depression, Screening, Referral pathways, Integrated services

## Abstract

**Background:**

Responding to and caring for women who experience mental health problems during the perinatal period, from pregnancy up to one year after birth, is complex and requires a multidisciplinary response. Family physicians are ideally placed to provide an effective response as it is recognised that they are responsible for organising care and supports for women and their families. This paper reports an integrative review undertaken to examine family physicians’ perceived role in perinatal mental health care and concludes with recommendations for health policy, research and practice.

**Method:**

A systematic search of literature in seven databases from January 2000 to March 2016 identified a total of 1125 articles. Qualitative, quantitative and mixed-method studies were eligible for inclusion if they explored family physicians’ experiences of caring for women who experience perinatal mental health problems.

**Results:**

Thirteen articles reporting 11 studies met the inclusion criteria for this review and quality of included studies were assessed using published criteria for the critical appraisal of qualitative and quantitative research methods. Cross-study narrative syntheses of quantitative and qualitative findings are presented under three themes: identification of perinatal mental health problems, management of perinatal mental health problems and barriers to care provision. While family physicians recognise their role in relation to perinatal mental health the collective interpretation revealed that; they receive variable levels of preparation for this role, no consistent approach to screening exists, pharmacological management of mood disorders is the main treatment modality and limited access to specialist perinatal mental health services exists which impacts on pharmacology decisions.

**Conclusion:**

Family physicians require timely access to local integrated care pathways that provide a wide range of services that are culturally sensitive, perinatal mental health specific, support psychological well-being and infant/family mental health. Family physicians are open to incorporating a brief validated screening tool into primary practice supported by succinct guidelines. Research that examines training needs in relation to perinatal mental health could be used to inform family physician training programmes and curriculum development around perinatal mental health.

## Background

The perinatal period from pregnancy through to the first year after birth, is recognised as a time of significant risk for development, relapse or recurrence of mental health problems [1, 2]. The term perinatal mental health problems (PMHPs) encompasses the full range of mental health disorders encountered by women in this period ranging from perinatal depression and anxiety to more serious perinatal mental health (PMH) issues including severe depression, bipolar disorder, psychosis, and posttraumatic stress disorder [1–3].

It is important to recognise and treat PMHPs across the diagnostic spectrum [4–6] as PMHPs may have significant consequences for the woman, her baby and family. Antenatal depression is associated with preterm birth and low birthweight [7]. Perinatal depression and anxiety are associated with negative outcomes for the developing fetus, child, adolescent [4, 6, 8–11] and partner relationship [9]. In the primary care setting, family physicians (FP’s) are particularly well-placed to assume a leading role in the management of PMHPs based on their role as the primary care provider [12, 13]. In this context, FP’s have; knowledge of a woman’s general wellbeing and mental health history, ongoing contact with mothers, infants and families throughout the perinatal period and liaise with primary and specialist mental health services [12, 13]. Current policy and practice guidance largely overlook the role of FPs in supporting women with mental health issues during the perinatal period [13]. Furthermore, the need to synthesise healthcare professionals (HCPs) experiences of providing care to women with PMHPs and to triangulate findings with the synthesis of women’s experiences has been identified [14]. A systematic review [15] examined quantitative studies on FPs recognition and management of perinatal depression and anxiety. Similarly, a meta-synthesis explored the diagnosis and management of perinatal depression and anxiety in general practice [16]. This current review synthesises the findings from qualitative and quantitative studies to provide a comprehensive review of the global evidence exploring FPs role in PMH. To this end, an integrative review of qualitative, quantitative and mixed-method studies on FP’s experiences of caring for women who experience PMHPs was conducted. Within this review, the term FP is used and incorporates the term general practitioner (GP) which is the term used in the Republic of Ireland, United Kingdom, Australia and Commonwealth countries.

## Methods

This review was guided by Whittemore and Knafl’s [17] integrative review methodology framework, which combines findings from qualitative and quantitative research on a specific subject to provide an all-encompassing understanding of the review question. The review was informed by the modified MOOSE standards [18] for reporting systematic reviews of observational research and reported across Whittemore and Knafl’s [17] five stage framework; problem identification, literature search, data evaluation, data analysis and presentation of findings.

### Problem identification

The aim of this integrative review was to explore the evidence relating to FP’s perceptions and experiences of caring for women who experience PMHPs to develop practical learning points that can be applied to healthcare professional training programmes and inform practice, research, education and policy developments.

### Objectives

The objectives of the review were to systematically identify, select, critically appraise and synthesise studies that examine FPs’ perceptions and experiences of caring for women who experience PMHPs.

### Literature search

Medical subject headings (MeSH), specific database headings, thesaurus and key words were used in conjunction with Boolean operators, truncation and synonyms (Table 1) to search seven electronic databases from January 2000 to March 2016. Databases searched included: Medline, EMBASE, Cumulative Index to Nursing and Allied Health Literature, PsycINFO, Cochrane, SCOPUS, Web of Science. The search was piloted in MEDLINE and CINAHL and individually adapted to each database.

**Table 1:** Search Terms

Search Terms	
“family practi^*^” OR “family physician” OR “family practice” OR “physicians, family” OR “primary health care” OR “physicians, primary care” OR “family doctor” OR “general practi^*^” AND “mental disorder” OR “adjustment disorder” OR “affective disorder” OR “dysthymic disorder” OR “mood disorder” OR psychiat^*^ OR “behaviour control” OR “psychological phenomena” OR depression OR “mental health” OR “stress disorder” OR “anxiety disorder” OR “maternal welfare” OR “maternal health” OR “mental hygiene” OR bipolar OR “obsessive compulsive disorder” OR psychosis OR “psychological distress” OR “somatic disorder” OR “somatoform disorder” OR “mental illness” OR “emotional distress” OR “emotional care” OR “maternal distress” OR “psychosocial wellbeing” OR PTSD OR OCD AND antenatal OR antepartum OR prenatal OR pregnancy OR perinatal OR postnatal OR postpartum OR puerperal.	

Qualitative, quantitative and mixed-method studies published in peer-reviewed journals that researched FP’s experience of caring for women with PMHPs were eligible for inclusion. A 15 year timescale was chosen to ensure a comprehensive coverage of contemporaneous relevant literature given the increasing emphasis on PMH during this timeframe.

Results of database searches identified 1125 articles, which were exported to EndNote reference management system. Duplicates were removed (Endnote and manually) resulting in 971 articles. Titles and abstracts were screened by MN for relevance based on inclusion criteria and discussion with the research team and 25 were forwarded for full text evaluation (MN and OD).

### Data extraction and evaluation of data

**Fig. 1:**
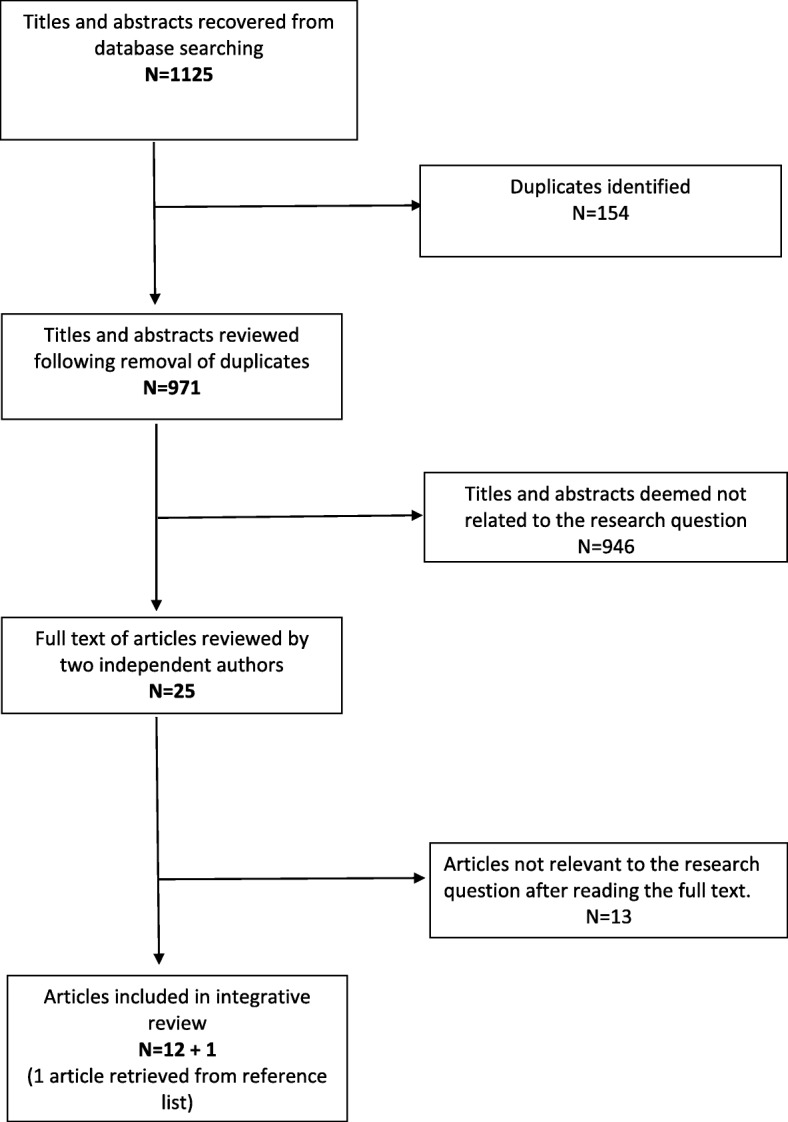
Prisma Flow Diagram

Evaluation of the 25 full text articles comprised of two levels of assessment. The first level assessment involved removal of articles based on inclusion and exclusion criteria. Twelve studies met the inclusion criteria. The reference lists of included studies were examined and one additional study was identified [19] resulting in 13 studies for review, comprising 5 qualitative studies and 8 quantitative studies (Fig. 1). Data were extracted on study aim, design, sample strategy and size, data collection method, analytical approach, strengths and limitations and key findings (Table 2).

**Table 2:** Descriptive characteristics of studies included in the review

Title, Author, publication year and country	Study aim	Design	Sample strategy and sample size	Data collection method	Analytical approach	Strengths and /Limitations	Key findings reported by authors
Recognition and management of perinatal depression in general practice. Buist et al. (2005), Australia [28].	To identify ways to improve detection and access to treatment.	A cross- sectional survey.	A random sample of 1075 general practitioners (GPs). Response rate (*n* = 246, 22.9%). A convenience sample of 908 women. Response rate (*n* = 525, 57%).	Questionnaire (10 multi-choice questions) and vignette.	Descriptive and inferential statistics (Analysis of Variance).	Random sample of general practitioners (GPs). Low response rate of 22.9% but consistent with other GP study response rates. Reliability and validity of the questionnaire and vignette not reported.	GPs preferences for antidepressant medication (antenatally 77% and postnatally 97%) contrasted strongly to women’s preferences for antidepressant medication (antenatally 22% and postnatally 54%). Perceived barriers to all treatments included unavailable resources, time, language or beliefs, reluctance of women to disclose mental health issues and denial/non-acceptance by women.
Are family physicians appropriately screening for postpartum depression?. Seehusen et al. (2005), Washington [27].	To determine how frequently Washington state FPs screen for PPD, what methods they use to screen and what influences their screening frequency. To explore FPs’ beliefs, attitudes and feelings concerning PPD and what screening tools they use. To identify factors associated with increased screening.	Cross-sectional survey.	A Random sample of 594 FPs. Response rate (*n* = 362, 60.9%).	A 25-item questionnaire developed for the study. Pilot tested for face validity.	Frequencies (X^2^ analysis, Multiple logistic regression, Bivariate analysis).	Random sample of FPs. Good response rate of 60.9%. Respondents were recruited from the Washington Academy of Family practice, a professional society, where members may be more likely to be aware of and follow recommendations for screening. Questionnaire tested for face validity only. Women and younger physicians responded disproportionately to the survey which may have led to an over estimation of screening rates.	71% of FPs were always or often screening for postpartum depression (PPD) at routine postpartum gynaecologic visits and 46% at well child visits, with 30.6% using a validated screening tool and of those, 82% used a standardised clinical interview. A significant number of respondents believed that screening at every postpartum visit (19.2%) and well-child visit (34.9%) would take too much effort. A variety of tools are used to screen for PPD. Formal training on PPD was received from a variety of sources.
Health professional’s knowledge and awareness of perinatal depression: Results of a national survey. Buist et al. (2006), Australia [29].	To evaluate the extent to which perinatal mood disturbances are recognised.	A cross- sectional survey.	A random sample of 1075 GPs. Response rate (*n* = 246, 23%). A random sample of 610 Maternal Child Health Nurses. Response rate (*n* = 338, 55%) A random sample of 995 Midwives. Response rate (*n* = 569, 57%).	A 10-item knowledge questionnaire based on work of Watts and Pope (1998) and a depression vignette based on work of Jorm et al. (2000).	Descriptive and inferential statistics (ANOVA, t-tests).	Random sample of GPs. Low response rate of 22.9%. Reliability and validity of the questionnaire and vignette not reported.	GPs had similar positive awareness scores for perinatal depression compared to both midwives and maternal child health nurses. Depression more likely to be considered postnatally. In relation to the vignette GPs were more likely than MCHNs and midwives to provide an accurate diagnosis (91.1% v 81.7% and 79.3% respectively). The greatest difference among health professionals was in the use of antidepressants with GPs being significantly more likely to choose these in comparison to MCHNs or Midwives (95% CI 8.4–23.2 and 20.9–34.3 respectively).
GPs’ and health visitors’ views on the diagnosis and management of postnatal depression: a qualitative study. Chew-Graham et al. (2008), UK [30].	To explore the views of GPs and health visitors (HV) on the diagnosis and management of postnatal depression.	A qualitative study nested within a multicentre randomised controlled trial (RESPOND trial). (Underpinning methodological approach not identified).	Purposive sample. GPs (*n* = 19). HVs (*n* = 14).	In-depth, semi-structured interviews.	Thematic analysis (Strauss 1986).	Nineteen GPs participated in the study however data saturation, informed consent and relationship between researcher and participants were not addressed.	Psychosocial aetiology was attributed to the cause of PPD and ambivalence about the status of PPD as a separate condition was identified. GPs relied on instinct or clinical intuition to alert them to the possibility of PPD. There was a reluctance to actively look for PPD or label a woman with PPD because of lack of referral options available. GPs identified the health visitor as a support for the woman.
Primary Care Physicians’ Beliefs and Practices toward Maternal Depression. Leiferman et al. (2008), USA [19].	To better understand and identify potential differences in attitudes, beliefs, efficacy, practices and current barriers (i.e. patient, physician and system) toward managing maternal depression across primary care specialities.	Cross-sectional survey.	A convenience sample of 971 primary care providers (PCPs). Response rate (*n* = 232, 23.9%). Response rate Obstetricians (*n* = 49, 22.6%), Paediatricians (*n* = 81, 37.3%) and family medicine practitioners (*n* = 87, 40.1%).	60-item questionnaire developed for the study (web or mail). Content validity by expert panel and pilot tested.	Descriptive and inferential statistics (Chi-square and one-way ANOVAs).	Convenience sample with response rate of 40.1% (*n* = 87). Reliability of questionnaire not determined.	Screening: 29.9% of family medicine physicians never/rarely assessed for maternal depression and 70.1% screened monthly/weekly/daily. The majority of family medicine physicians treat maternal depression by prescribing medication (92%) followed by referral to the mental health specialist off-site (82.8%) and 70.1% provide counselling in office and 37.9% refer to community support groups. The most commonly reported barriers that reduce the likelihood of screening and treatment for depression across specialities were limited time, patient barriers (perception that patient was unwilling to talk about mental health issues and the perception of stigma), lack of knowledge and skills and responsibility for follow-up care.
Disclosure of symptoms of postnatal depression, the perspectives of health professionals and women: a qualitative study. Chew-Graham et al. (2009), UK [31].	To explore GPs, health visitor’s and women’s views on the disclosure of symptoms which may indicate postnatal depression in primary care.	A qualitative study nested within a multi-centre pragmatic randomised controlled trial (RESPOND trial). (Underpinning methodological approach not identified).	Purposive sample. GPs (*n* = 19). HVs (*n* = 14). Women (*n* = 28).	In-depth, semi-structured interviews.	Thematic analysis (Strauss 1986).	Nineteen GPs participated in the study however data saturation, informed consent and relationship between researcher and participants were not addressed.	GPs were reluctant to use the label PPD with women because of the stigma that they perceived women felt and the effect this would have on the consultation and because they felt women would recover without formal interventions. A lack of user-friendly health services or referral options, limited appointment availability, lack of continuity of care and feeling antidepressants were the only treatment options were identified as barriers to management. GPs identified offering a return visit as a strategy to support women presenting with PND.
Depression during pregnancy: views on antidepressant use and information sources of general practitioners and pharmacists. Ververs et al. (2009), The Netherlands [22].	To investigate whether GPs and pharmacists in the Netherlands obtain information on the safety of gestational drug use and the pharmco-therapeutic approach when managing depression and anxiety during pregnancy.	Cross-sectional survey.	A random sample of 700 GPs and 700 pharmacists. Response rate GPs (*n* = 130, 19%). Pharmacists (*n* = 144, 21%).	20 - item Questionnaire developed for the study.	Descriptive and inferential statistics (chi-squares tests).	Random sample of GPs. Low response rate of 19%. Reliability and validity of the questionnaire not reported.	GPs consulted a variety of sources for information on drugs during pregnancy. Variable practices in relation to referral were identified with 29% of GPs in this study never referring a woman who is pregnant and on anti-depressants to a psychiatrist and 50% some-times refer. The main reason for treating depression or anxiety during pregnancy was because the severity of maternal complaints outweigh possible risks for the child (*n* = 124). A lack of knowledge was evident around the consequences of perinatal depression.
Falling through the net- Black and minority ethnic women and perinatal mental healthcare: health professionals’ views. Edge (2010), UK [32].	To investigate health professionals’ views about perinatal mental healthcare for Black and minority ethnic women.	Qualitative study (Underpinning methodological approach not identified).	Purposive sample of 42 healthcare professionals. Third sector (Focus group, *n* = 3). Specialist midwives (in-depth interviews, *n* = 2). Hospital midwives (Focus group, *n* = 9). Community midwives (Focus group, *n* = 11). Midwifery managers (Focus group, *n* = 5). GPs (In-depth interview, *n* = 5). Health visitor (Focus groups, *n* = 5). Hospital doctor (in-depth interview, 2).	In-depth, semi-structured interviews.	Framework analysis (Ritchie et al. 1994).	Five GPs participated in this study. Data saturation, informed consent and relationship between researcher and participants were not addressed. Appropriate data verification strategies were identified.	Perinatal depression was not routinely screened for during antenatal and postnatal visits to the GP. It was acknowledged that postnatal depression in women from black and minority communities was rarely diagnosed and may be missed. GPs appeared highly resistant to using validated screening tools and valued intuition to identify women with current symptoms of PMHPs in preference to screening tools. Lack of confidence, competence and training in identifying and managing perinatal mental health problems irrespective of ethnic or cultural backgrounds was reported. Lack of cultural competence in services, timely access to appropriate care and the absence of clearly defined care pathways were identified as barriers to the provision of effective perinatal mental healthcare.
Primary care physician’s attitudes and practices regarding antidepressant use during pregnancy: a survey of two countries. Bilszta et al. (2011) Canada and Australia [23].	To explore primary care physician’s beliefs and practices toward perinatal depression by investigating the knowledge, attitudes and practices affecting a physician’s decision to continue or discontinue a woman’s antidepressant medication during this period.	A cross- sectional survey.	A convenience sample of 188 primary care physician from Australia (GPs (77)) and Canada (FPs (111)). Response rate Australian GPs (*n* = 61, 79.2%). Canadian FPs (*n* = 35, 31.5%).	Questionnaire developed for the study.	Descriptive and inferential statistics (Chi-square test of association with Fisher’s exact test).	Different sampling strategies used for different populations. Convenience sample with response rate of 79.2% (Australian GPs) and 31.5% (Canadian FPs). Australian GPs were attending training workshops about identification, treatment and management of depression and were a self-selected sample. Reliability of questionnaire not determined.	Perceived levels of misinformation about the safety of antidepressant medication in pregnancy, belief that pregnant depressed women should be treated differently from non-pregnant depressed women, concerns over the legal liability and patient concerns influence prescribing practices for GPs and family physicians.
Antidepressants for mothers: What are we prescribing? Kean et al. (2011) Scotland [34].	To investigate current prescribing practices among GPs of antidepressants to mothers presenting in first trimester of pregnancy and during breastfeeding.	A cross-sectional survey.	A convenience sample of 78 GPs. Response rate (*n* = 32, 41%). (methodological approach not clear).	Questionnaire (two vignettes) developed for the study.	Microsoft excel. Descriptive statistics.	Convenience sample with response rate of 41% (*n* = 32). Reliability and validity of questionnaire not determined.	One in four GPs (*n* = 8) recommended a class of antidepressants rather than a specific drug. One in ten GPs preferred not to prescribe an antidepressant and one in four would avoid ‘all drugs’. Reasons for avoiding antidepressants included lack of practitioner experience (*n* = 7), higher teratogenicity risk (*n* = 5) and lack of data (*n* = 4).
A qualitative study into how guidelines facilitate general practitioners to empower women to make decisions regarding antidepressant use in pregnancy. McCauley and Casson (2013), Northern Ireland [33].	To develop an in-depth understanding of GPs’ experience of using guidelines in the treatment of perinatal depression and if this enabled them to empower women to become involved in treatment decisions.	Qualitative study (Underpinning methodological approach not identified).	Purposive sample of GPs (*n* = 8).	In-depth, semi-structured interviews.	Colaizzi’ (1978) process of analysis.	Eight GPs participated in this study. Data saturation was not addressed. One data verification strategy (verification of themes between the chief investigator and researcher) was identified.	GPs reported low usage of guidelines. Treatment decisions involved balancing the impact of the severity of symptoms with the possibility of adverse effects of antidepressants on the fetus and timing of treatment. GPs acknowledged the support available from the local mental health team and voluntary organisations. A lack of specific, available resources, specialists’ perinatal mental health services, delays in response due to lengthy appointment waiting lists and increasing workloads were identified as barriers to complicated treatment decisions.
Postpartum depression: the (in) experience of Brazilian primary healthcare professionals. Santos et al. (2013), Brazil [36].	To describe primary healthcare physicians’ and nurses’ knowledge and experience in screening and treating women with postpartum depression.	Qualitative descriptive.	Purposeful sample. Physicians (*n* = 7). Nurses (*n* = 10).	In-depth, semi-structured interviews. Observation of contacts between HCPs and postpartum women –observation guide developed for study.	Inductive content analysis (Hsieh and Shannon 2005).	Seven physicians participated in this study and the researchers discussed data saturation. Observations of contacts between HCPs and postpartum women supported data findings. Appropriate data verification strategies identified. The relationship between researcher and participants was not addressed.	Physician’s reported limited knowledge, awareness and recognition of PPD and had limited direct clinical experience of caring for women who experience PPD. The focus of care was on physical wellbeing. PPD were seen as the responsibility of psychiatrists in relation to identification, diagnosis and treatment. A lack of specific guidance, training, skills, time and resources were identified as barriers to the provision of care to women with perinatal mood disorders.
Primary Care Physicians’ Attitudes Toward Postpartum Depression: Is It Part of Their Job. Glasser et al. (2016) Israel [35].	Israeli primary care physicians’ attitudes and practice regarding postpartum depression (PPD).	Cross sectional survey.	A convenience sample of 345. Response rate 65% (*n* = 224 paediatricians and family practitioners). Family practitioners (*n* = 102). Paediatricians (*n* = 122).	Questionnaire developed for study.	Descriptive and inferential statistics (Chi-square).	Convenience sample with response rate of 65% (*n* = 224). Reliability and validity of questionnaire not determined.	Family practitioners identified the importance of being able to recognise the signs of PPD. While 84.6% of family practitioners would become somewhat involved to include clarifying the situation, keeping attentive, consulting with colleagues and/or referring the mother to another professional. 83% would be willing to use a brief questionnaire to identify women with signs of PPD.

The second level of assessment involved a critical appraisal (MN, RG and OD) to determine methodological quality of included studies. Due to the variety of methodologies and designs, two method-specific tools were identified to assess quality of evidence. For qualitative studies, the Critical Appraisal Skills Programme (CASP) [20] tool was used (Table 3) and the Rees et al. [21] survey checklist (Table 4) was utilised for cross-sectional studies. Each criterion was recorded as “Yes” or “No” or “Clear” or “Unclear” and results of appraisal were discussed between MN, OD and RG with discrepancies resolved by consensus. Overall studies were found to be of good methodological quality with qualitative studies meeting between seven and nine of the ten appraisal criteria (Table 3) and quantitative studies meeting between eight and thirteen of the fourteen appraisal criteria (Table 4). All studies identified research aims, justified the appropriateness of design, used well-defined sampling strategies, presented clear statements of findings and outlined the value of their research. In terms of the quantitative studies, response rates varied between 19% [22] and 79.2% [23] and only two studies attempted to explore non-responders. Studies were limited to a convenience sample in a specific geographic area (*n* = 4, Table 4). Eight studies did not provide sufficient information to appraise validity and reliability of measures (Table 4). Four qualitative studies were unclear regarding data saturation (Table 3) and three did not report an explicit statement of ethical approval or informed consent (Table 3). Four qualitative studies did not provide details of adequate consideration of the relationship between researcher and participants (Table 3).

**Table 3:** Methodological quality of qualitative studies

Authors	1	2	3	4	5	6	7	8	9	10	Total scores
Chew-Graham et al. (2008) [30]	Yes	Yes	Yes	Yes	Unclear^a^	Unclear	Unclear^b^	Yes	Yes	Clear	7/10
Chew-Graham et al. (2009) [31]	Yes	Yes	Yes	Yes	Unclear^a^	Unclear	Unclear^b^	Yes	Yes	Clear	7/10
Edge (2010) [32]	Yes	Yes	Yes	Yes	Unclear^a^	Unclear	Unclear^b^	Yes	Yes	Clear	7/10
McCauley and Casson (2013) [33]	Yes	Yes	Yes	Yes	Unclear^a^	Yes	Yes	Yes	Yes	Clear	9/10
Santos et al. (2013) [36]	Yes	Yes	Yes	Yes	Yes	Unclear	Yes	Yes	Yes	Clear	9/10

Keys:

1. Was there a clear statement of the aims of the research?

2. Is a qualitative methodology appropriate?

3. Was the research design appropriate to address the aims of the research?

4. Was the recruitment strategy appropriate to the aims of the research?

5. Was the data collected in a way that addressed the research issue?

6. Has the relationship between the researcher and participants been adequately considered?

7. Have ethical issues been taken into consideration?

8. Was the data analysis sufficiently rigorous?

9. Is there a clear statement of findings?

10. How valuable is the research?

Critical Appraisal Skills Programme [20]

^a^Theoretical saturation not discussed

^b^Did not explicitly discuss informed consent

**Table 4:** Methodological quality of quantitative studies

Study	1a	2a	2b	2c	2d	3a	3b	3c	4a	4b	5a	6a	7a	8a	Total score
Buist et al. (2005) [28]	Yes	Yes	Yes	Yes	No	Yes	Unclear	Unclear	Yes	Yes	Yes	Yes	Yes	Clear	11/14
Seehusen et al. (2005) [27]	Yes	Yes	Yes	Yes	Yes	Yes	Yes	Unclear	Yes	Yes	Yes	Yes	Yes	Clear	13/14
Buist et al. (2006) [29]	Yes	Yes	Yes	Yes	No	Yes	Unclear	Unclear	Yes	Yes	Yes	Yes	Yes	Clear	11/14
Leiferman et al. (2008) [19]	Yes	Yes	No^a^	Yes	Yes	Yes	Yes	Unclear	Yes	Yes	Yes	Yes	Yes	Clear	12/14
Ververs et al. (2009) [22]	Yes	Yes	Yes	No	No	Yes	Unclear	Unclear	Yes	Yes	Yes	Yes	Yes	Clear	10/14
Bilszta et al. (2011) [23]	Yes	Yes	No^a^	Yes	No	Yes	Unclear	Unclear	Yes	Yes	Yes	Yes	Yes	Clear	10/14
Kean et al. 2011 [34]	Yes	Unclear	No^a^	No	No	Yes	Unclear	Unclear	Yes	Yes	Yes	Yes	Yes	Clear	8/14
Glasser et al. (2016) [35]	Yes	Yes	No^a^	No	No	Yes	Unclear	Unclear	Yes	Yes	Yes	Yes	Yes	Clear	9/14

Keys:

A Are the results valid?

1. Objectives:

1a. Are the objectives of the study clearly stated?

2. Design:

2a. Is the study design suitable for the objectives?

2b. Did the subject represent the full spectrum of the population of interest?

2c. Has ethical approval been obtained?

2d. Were measures used to contact non-responders?

3. Measurement and observation

3a. Is it clear what was measured, how it was measured and what the outcomes were?

3b. Are the measurements valid?

3c. Are the measurements reliable?

B What are the results

Presentation of results

4a. Are the basic data adequately described?

4b. Are the results presented clearly, objectively and in sufficient detail to enable readers to make their own judgement?

Analysis

5a. Are the methods appropriate to the data?

C Will the results help locally?

6 Discussion

6a Are the results discussed in relation to existing knowledge on the subject and study objectives?

7 Interpretation

7a. Are the authors conclusions justified by the data?

8 Implementation

8a Can any necessary change be implemented in practice?

Rees et al. [21]

^a^convenience samples

### Analysis of data

Given the heterogeneity of included literature, thematic analysis of extracted findings of each study was undertaken [24] because of its potential to draw conclusions based on common elements [25]. The steps used to conduct thematic analysis were guided by Lucas et al. [25] and Smith et al. [26].

### Presentation of results/findings

Table 2 displays the characteristics of the included studies. Studies were conducted in the USA [19, 27], Netherlands [21], Canada [23], Australia [23, 28, 29], UK [30–33], Scotland [34], Israel [35] and Brazil [36]. All eight quantitative studies were cross-sectional in nature. Sample sizes varied across these studies and ranged between 32 to 362 respondents. Qualitative studies consisted of one qualitative descriptive [36] and four broad qualitative studies [30–33]. Qualitative study sample sizes ranged between 5 [32] and 19 [30–32] participants. Four articles reported on results from two studies [28, 29] and [30, 31] however, authors reported different aspects of findings in each of these articles. Five studies focused on PPD [27, 30, 31, 35, 36], three examined FP’s recognition and management of perinatal depression [19, 28, 29] one study explored PMH [32] and four studies focused on the use of antidepressants [22, 23, 33, 34].

## Results

**Fig. 2:**
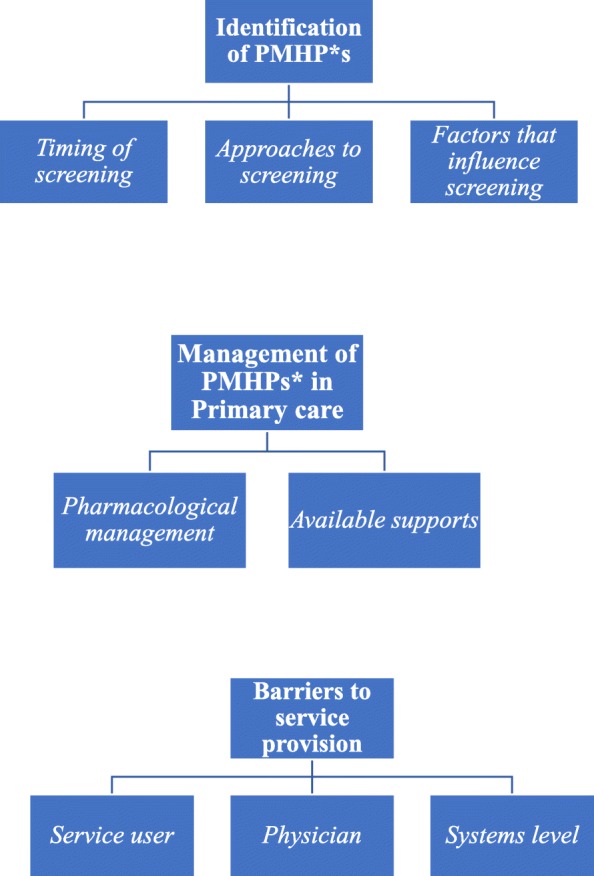
Themes and sub-themes

The findings of the review are presented under three main themes generated through analysis: identification of PMHPs, management of PMHPs in primary care and barriers to care provision. These broad themes contain a number of sub-themes as illustrated in Fig. 2 and Table 5 contains excerpts from the original studies to support these findings.

**Table 5:** Excerpts from original studies

Buist et al. (2005) [28]	Knowledge and awareness Anti -depressants Barriers Interventions Barriers to screening- time Referrals	Mean knowledge score out of 100 was 66. General practitioners (GPs) had significantly higher positive awareness of perinatal depression (7.1, SD:2.7) and corresponding low negative awareness (− 0.2, SD: 2.3) compared with the postnatal women surveyed in this study (4.0, SD:3.5 and 1.1, SD: 1.7) (*p* < 0.0001). In response to the vignette GPs preferences for antidepressant medication (antenatally 77% and postnatally 97%) contrasted strongly to women’s preferences for antidepressant medication (antenatally 22% and postnatally 54%). Women’s preferred treatment options were for natural remedies in comparison to GPs preference for antidepressant medication. Perceived barriers to all treatments included unavailable resources (47%), family language or beliefs (23%), reluctance of patient (19%), None (18%), Financial (12%), denial/non-acceptance of patient (12%) and community attitudes (6%). GPs beliefs around the usefulness of interventions for perinatal depression identified antidepressant medication as a third choice behind counselling and partner support for the woman. Time was identified as the main negative impact of treating depression. GPs reported making referrals to mother –baby unit (68%), counsellor (69%) and psychiatrist (85%), midwife (42%), telephone/crisis line (12%), naturopath (3%).
Seehusen et al. (2005) [27]	Time of screening Screening tools Training Factors influencing screening Barrier to screening - time	The majority of family physicians (FPs) were screening at routine postpartum gynaecologic visits but not at well child visits. A variety of tools are used to screen for postpartum depression (PPD). 30.6% used a validated tool. The standardised clinical interview was used by the majority of those who screen (82%) followed by The Beck Depression Inventory questionnaire type tool (29%). Formal training on PPD was received in a variety of venues (residency, medical literature and through continuing medical education conferences) Being female, belief that PPD is common enough and serious enough to warrant screening, training in PPD during residency and medical literature review and disagreement that screening takes too much effort were significantly associated with more frequent screening at postpartum gynaecological visits and well-child visits. A significant number of respondents believed that screening at every postpartum visit (19.2) and well-child visit (34.9%) would take too much effort.
Buist et al. (2006) [29]	Awareness Diagnosis Antidepressants	GPs had similar awareness scores for perinatal depression compared to both midwives and maternal child health nurses. Depression more likely to be considered postnatally. In relation to the vignette GPs were more likely than MCHNs and midwives to provide an accurate diagnosis (91.1% v 81.7% and 79.3% respectively) The greatest difference among health professionals was in the use of antidepressants with GPs being significantly more likely to choose these rather than MCHNs or Midwives (95% CI 8.4–23.2 and 20.9–34.3 respectively)
Chew-Graham et al. (2008) [30]	Conceptualisation of postnatal depression Screening Referral options Treatment strategies Support strategies Responsibility	Psychosocial aetiology was attributed to the cause of postnatal depression (PND) and ambivalence about the status of PND as separate condition was identified. GPs relied on instinct or clinical intuition to alert them to the possibility of PND. There was a reluctance to actively look for PND or label a woman with PND because of lack of referral options available. GPs used a variety of strategies to care for women and described how the label they used for the woman’s problems determined what management strategies they employed. GPs identified the Health visitor as a support for the woman however some GPs reported observing an unwillingness of some health visitors to care for women with postnatal depression. National policy and local organisations changes impacted on care with no one health professional assuming overall responsibility for the care of women with postnatal depression.
Leiferman et al. (2008) [19]	Responsibility Confidence Screening Prescribing medication Counselling/referral Community support groups Barriers to screening Knowledge and skills Responsibility Screening tool Referral Barriers to treatment Training	Family medicine physicians were most likely to feel responsible for and confident in treating maternal depression in comparison to obstetricians and paediatricians. Screening: 29.9% of family medicine physicians never/rarely assessed for maternal depression and 70.1% screened monthly/weekly/daily. Use of screening tools: mean 2.40 (SD 0.89). 92% of family medicine practitioners typically treat maternal depression by prescribing medication followed by referral the MH specialist off-site (82.8%) and 70.1% provide counselling in office and 37.9% refer to community support groups. The most commonly reported barriers that reduce the likelihood of screening for depression across specialities were limited time, patient barriers (perception that patient was unwilling to talk about mental health issues and the perception of stigma), lack of knowledge and skills and responsibility for follow-up care. Over 90% of family medicine physicians reported a willingness to implement a screening tool and to place a two-item tool on an intake form. Referral: 62.8% reported never/rarely referring patients for treatment for maternal depression. The most commonly reported barriers to treatment of maternal depression across specialities were limited time, lack of knowledge and skills and responsibility for follow-up care and liability issues. Training: overall PCPs perceived mental health resources to be inadequate. Over 90% of PCPs expressed a willingness to learn about ways to enhance patient communication about mental health issues. More training on mental health issues in the form of continuing education units, guidelines, seminars, workshops and computer deliverables was desired across PCPs.
Chew-Graham et al. (2009) [31]	Conceptualisation of PND Screening Label Stigma Services Referral options Antidepressants Label Support strategy Barriers to disclosure Barrier to care provision	Psychosocial aetiology was attributed to the cause of postnatal depression and ambivalence about the status of postnatal depression as separate condition as compared with depressive illness at other times in a woman’s life was identified. GPs relied on instinct or clinical intuition to alert them to the possibility of PND. There was a reluctance to actively look for signs of PND or use screening instruments. GPs were reluctant to use the label for PND with women because of the stigma that they perceived women felt and the effect this would have on the consultation and because they felt women would recover without formal interventions. Other reasons identified were a lack of services or referral options and feeling antidepressants were the only treatment options. However, other GPs describe consultations where women were happy to accept the label PND. GPs identified offering a return visit as a strategy to facilitate a discussion and support women presenting with PND. However, they identified barriers that hinder disclosure including not user-friendly health services and limited appointment availability. Some GPs reported consciously inhibiting disclosure in order not to be placed in the position of addressing PND. Lack of continuity of care was identified as a barrier to care provision.
Ververs et al. (2009) [22]	Guidelines Treatment decisions- pharmacists Sources of information on antidepressant use in pregnancy Guidelines Manufacturers of specific drugs Internet Education Referral Treatment decision Antidepressants Treatment decision Management Antidepressant Psychotherapy Knowledge	Only one GP had access to a local written policy on the treatment of depression and anxiety during pregnancy. Almost three quarters of GPs regularly consult pharmacists for information on drugs during pregnancy. The reference used most frequently by GPs is the “Pharmacotherapy Compass” The Dutch National Health Insurance System Formulary issued annually in the Netherlands. Guidelines on the treatment of depression (not specific to pregnancy) issued by the Dutch College of General Practitioners are used to a lesser extent. A quarter of GPs contact the manufacturer of a specific drug for information. 45% use the internet to look for information on scientific evidence or reports from consensus groups. GPs use different sources of information on antidepressant use in pregnancy. One in five answered yes to the question of whether the subject “treatment of depression and anxiety during pregnancy” has been covered during professional education courses. Referral: 29% of GPs in this study never refer a woman who is pregnant and on anti-depressants to a psychiatrist and 50% refer sometimes. 9% of GPs state that they sometimes advice terminating the pregnancy when a woman who uses antidepressants becomes pregnant. 55% of GPs never advised substituting psychotherapy for medication in order to prevent drug exposure to the child. The main reason for treating depression or anxiety during pregnancy was because the seriousness of maternal complaints outweighs possible risks for the child (*n* = 124). Reasons for avoiding antidepressants during pregnancy were because antidepressants may have negative effects on the unborn child (*n* = 93), withdrawal symptoms after birth (*n* = 44) not officially registered for use during pregnancy (*n* = 39), perceptions that psychotherapy is as effective as antidepressants (*n* = 36). Large differences in views on the pharmacological management of depression before and during pregnancy reported. A varied pattern of antidepressant use was reported. Most respondents underestimated the lasting effects of psychotherapy. A lack of knowledge was evident around the consequences of Perinatal depression with only 20% of GPs recognising the negative effects of depression and anxiety on a child’s development and on the management of perinatal depression.
Edge (2010) [32]	Diagnosis Screening Screening tools Lack of confidence, competence Training Barrier to provision of care Care pathways Lack of confidence in Multi-agency team members Relationships Diagnosis Conceptualisation of PND Cultural competence Awareness Language barriers	Acknowledgement that postnatal depression in women from black and minority communities was rarely diagnosed and may be missed. GPs privileged intuition over instrumentation did not routinely screen for PND, and appeared highly resistant to using validated psychiatric measures or screening tools such as the EPDS and PHQ-9. Lack of confidence, competence and training in identifying and managing perinatal mental health problems irrespective of ethnic or cultural backgrounds was reported. Lack of timely access to appropriate care and the absence of clearly defined care pathways identified as barriers to the provision of effective perinatal mental healthcare. Unfamiliarity between multi-agency team members generated lack of confidence in colleagues’ professional competence (linked to NHS reforms where HVs were moved out of general practice and into centralised services). The importance of establishing trusting relationships with Black women to support diagnosis of perinatal depression was identified. It was acknowledged that Black Caribbean women’s psychological responses were linked to their cultural identify in ways that made it difficult for them to ask for and receive help either from health professionals or from social/family resources. Lack of cultural competence in services acted as a barrier to detection of perinatal depression. Lack of awareness of culturally specific issues and some staff appeared to adopt a ‘colour-blind’ approach to caring for women from diverse ethnic groups instead concentrating on language barriers.
Bilszta et al. (2011) [23]	Factors influencing Prescribing practices Confidence-treatment decision	Perceived levels of misinformation about the safety of antidepressant medication in pregnancy, belief that pregnant depressed women should be treated differently from non-pregnant depressed women, concerns over the legal liability and patient concerns influence prescribing practices with GPs and family physicians reportedly feeling hesitant to prescribe, tapering dosages rather than discontinuing medication (continuation or discontinuation of use of antidepressants in pregnancy). The authors conclude that primary care physicians are not confident about the decision to treat pregnant women with antidepressants.
Kean et al. (2011) [34]	Antidepressants Factors influencing prescribing practices in pregnancy Factors influencing prescribing practices in breastfeeding Sources of information on antidepressant use in pregnancy and breastfeeding	One in four GPs (*n* = 8) recommended a class of antidepressants rather a specific drug. One in ten GPs (*n* = 3) preferred not to prescribe an antidepressant and one in four would avoid ‘all drugs’. The main reasons for choosing antidepressants in the first trimester of pregnancy were practitioner experience of drug (*n* = 12) and low teratogenicity (*n* = 10) and perception of drug safety (*n* = 7). Reasons for avoiding antidepressants included lack of practitioner experience (*n* = 7), higher teratogenicity risk (*n* = 5) and lack of data (*n* = 4). The main reasons for choosing antidepressants for women who were breastfeeding included drug safety (*n* = 11), practitioner experience of drug (*n* = 9) and low levels of antidepressants in breast milk (*n* = 5). Reasons for avoiding antidepressants in breastfeeding included excreted in breast milk (*n* = 7), lack of data (*n* = 3) and lack of practitioner experience (*n* = 3). The main source of information consulted in pregnancy was the British National Formulary (BNF) followed by specialist advice and in breastfeeding the BNF followed by manufacturer’s advice.
McCauley and Casson 2013 [33]	Lack of time Guidelines Treatment decisions Service user involvement in decisions Barrier to care provision Service user involvement in decisions Treatment decisions Antidepressants Factors influencing treatment practices Support referral options Barriers Service user involvement in decisions	GPs reported low usage of guidelines in practice due to lack of time and the volume of available guidelines. GPs acknowledged that guidelines provide best practice advice, a professional reference point and can be used as a defence against litigation in case of adverse reactions however, guidelines were also identified as generic, lacked specific and clear direction on treatment in the perinatal period, were restrictive and may inhibit flexibility and knowledge resulting in patient need not being met. GPs relied on their own professional experience and knowledge of the individual woman to make complex risk-benefit treatment decisions. Individualised information provision communicated using lay language in both written and verbal formats encouraged women to be involved in the decision –making process. Lack of specific or accurate guidance was described as a barrier to information provision and led to under treatment of pregnant women in general practice. Professional experience was used to determine the level of involvement that women wanted in the decision-making process. Treatment decisions involved balancing the impact of the severity of symptoms with the possibility of adverse effects of antidepressants on the foetus and timing of treatment. Female GPs acknowledged that their personal experience of pregnancy affected decisions. Lack of consultation with GPs by women led to abrupt stopping of antidepressants. GPs acknowledged the support available from the local mental health team and voluntary organisations. However, a lack of available resources, specialists’ perinatal mental health services, delays in response due to lengthy appointment waiting lists and increasing workloads were identified as barriers to complicated treatment decisions. GPs view the involvement of women in treatment decisions as central to women’s empowerment but clinical complexities and the level to which women want to be involved in decisions about medications in pregnancy limit involvement.
Santos et al. (2013) [36]	Knowledge and awareness Conceptualisation of PPD Guidelines Focus on physical wellbeing Responsibility Barriers to provision of care – training, skills, time, resources Lack of comfort Lack of space	Family physician’s in a city in Brazil reported limited knowledge, awareness and recognition of PPD and had limited direct clinical experience of caring for women who experience PPD. They viewed PPD as an uncommon problem attributed to hormonal changes. The clinical practice protocols available to physicians did not recommend any particular approach to perinatal mood disorders. The focus of care was on physical wellbeing. PPD was seen as the responsibility of psychiatrists in relation to identification, diagnosis and treatment. A lack of training, skills, time and resources were identified as barriers to the provision of care to women with perinatal mood disorders. Two challenges identified were a lack of comfort in approaching women who could potentially be experiencing PPD and lack of physical space for women to be treated.
Glasser et al. (2016) [35]	Responsibility Recognition of signs Management Referral Screening	The majority of family practitioners identified the importance of being able to recognise the signs of PPD. 84.6% of family practitioners would become somewhat involved to include clarifying the situation, keeping attentive, consulting with colleagues and/or referring the mother to another professional. 91.2% would be willing to use a brief questionnaire to identify women with signs of PPD.

### Identification of PMHPs

#### Identification of PMHPs

The theme identification of PMHPs explores timing of screening, approaches to screening and factors that influence screening practices.

### Timing of screening

The timing of screening was identified in three studies. In one study [27], 71% of FP’s screened often or always at routine postpartum gynaecologic visits and 46% at well child visits. Furthermore, 70.1% of the respondents in a second study [19] reported that they screened women for PMHPs monthly/weekly/daily. However, 29.9% reported never/rarely assessing for maternal depression. On the contrary, in the third study FPs reported not routinely screening for PPD [32] (Table 6).

**Table 6:** Screening tools identified within studies

Study	Screening instrument	Timing
Buist et al. (2005) [28]	The Edinburgh Postnatal Depression scale (EPDS)	Postpartum.
Seehusen et al. (2005) [27]	A standardised clinical interview (82%). The Beck Depression Inventory (29%). EPDS (10%). Zung Depression Scale (8%). Postpartum Depression Checklist (8%).	31% of family physicians (FPs) always screened for postpartum depression (PPD) at routine postpartum gynaecologic visits. 40% of FPs often screened for PPD at routine postpartum gynaecologic visits. 5.7% of FPs never screened for PPD at routine postpartum gynaecologic visits. 13% of FPs always screened for PPD at routine well child visits. 33% of FPs often screened for PPD at routine well child visits. 15.2% of FPs never screened for PPD at well child visits.
Buist et al. (2006) [29]	The Edinburgh Postnatal Depression scale (EPDS).	Postpartum.
Chew-Graham et al. (2008) [30]	Instinct or clinical intuition to alert GPs to the possibility of PPD.	Intuitional use - “So I’m not saying I would actually look for it, but I am hoping my antennae would tell me if there was a problem” (GP, M5, P.171).
Leiferman et al. (2008) [19]	Evidence of screening tool utilised by participants but screening tool not identified. Use of screening tool: mean 2.40 (SD = 0.89). Over 90% of family medicine physicians reported a willingness to implement a screening tool and to place a two-item tool on an intake form.	70.1% screened monthly/weekly/daily. 29.9% never/rarely assessed for maternal depression.
Chew-Graham et al. (2009) [31]	Instinct or clinical intuition to alert GPs to the possibility of PPD.	Intuitional use where a degree of suspicion is present.
Ververs et al. (2009) [22]	None identified.	N/A
Edge (2010) [32]	GPs privileged intuition over instrumentation and did not routinely screen for PPD and appeared highly resistant to using validated psychiatric measures or screening tools such as the EPDS and Patient Health Questionnaire (PHQ-9).	Infrequent based on intuition - “I am largely responsible for PHQ-9 being introduced…when it comes to my own type of practice, I very rarely get the PHQ-9 out and get people to tick boxes but I will take the questions from it and I will use those. So, umm, I would be lying if I said I used a formal structured questionnaire to get a clinical diagnosis, because I don’t” (GP1, P.19).
Bilszta et al. (2011) [23]	None identified	N/A
Kean et al. (2011) [34]	None identified	N/A
McCauley and Casson (2013) [33]	None identified	N/A
Santos et al. (2013) [36]	No evidence of screening tools used by primary healthcare professionals within the study region.	N/A
Glasser et al. (2016) [35]	No screening tools identified within the study. However, 91.2% of family practitioners indicated they would be willing to use a brief questionnaire to identify the signs of PPD.	N/A

### Approaches to screening

There was no consistent approach to screening for PMHPs. Screening focused on PPD with limited evidence of screening for anxiety or any other PMHP. A range of screening tools were used by FPs to screen for PPD (Table 6). A reluctance of FP’s to use screening instruments or actively enquire about symptoms of PPD was identified with FP’s relying on instinct or clinical intuition to alert them to the possibility of PPD [30, 31]. Similarly, FP’s appeared highly resistant to using validated screening tools and instead “privileged intuition over instrumentation” [32]. However, 83% of FP’s [35] reported that they would be willing to use a brief questionnaire to identify women with signs of PPD. Similarly, over 90% of FP’s [19] reported a willingness to implement a validated two-item screening tool.

### Factors that influenced screening

Factors associated with more frequent screening included the FP being female, knowledge of the prevalence and morbidity associated with PPD, training in PPD during residency and through evidence from medical literature review [27]. Some FP’s reported consciously inhibiting disclosure if they did not have access to referral pathways and they felt that women would recover without formal interventions [30, 31]. The importance of establishing trusting relationships with women to support screening and diagnosis of perinatal depression was identified [32].

#### Management of PMHPs in primary care

This theme explores strategies that FP’s instigate to care for women who experience PMHPs under the two subthemes of pharmacological management and available supports.

### Pharmacological management

Across studies, pharmacological management of PMHPs was identified as the main treatment modality offered to women in primary care. In response to the vignette [28], FP’s preferences were for antidepressant medication (antenatally 77% and postnatally 97%) which contrasted strongly to women’s preferences for antidepressant medication (antenatally 22% and postnatally 54%). FP’s were also significantly more likely to choose antidepressant medication than Maternal Child Health Nurses (MCHNs) and midwives (95% CI 8.4–23.2 and 20.9–34.3 respectively) [29]. However, when FP’s were asked about their beliefs around the usefulness of interventions for perinatal depression, they identified antidepressant medication as a third choice behind counselling and partner support for women [28]. Similarly, 92% of FP’s [19] typically treat maternal depression by prescribing medication followed by referral (82.29%) to the mental health specialist off-site and 70.1% provide counselling in office. However, in another study one in ten FPs surveyed [34] preferred not to prescribe antidepressants and one in four FPs would avoid ‘all drugs’. Reasons for avoiding antidepressants were lack of practitioner experience (*n* = 7), high teratogenicity risk (*n* = 5) and lack of data (*n* = 4). The contrasting findings may be related to the use of different questionnaires in the studies that examined FPs perceptions on perinatal antidepressant treatment. FPs were reluctant to identify PPD when the only course of action they felt available to them was prescribing antidepressants [31]. Similarly, FPs [33] viewed medication as a necessity rather than a choice because of the limited availability of referral options. The main reason for treating depression or anxiety during pregnancy was that the seriousness of maternal complaints outweighed possible risks for the child [22]. Treatment decisions involved balancing the impact of the severity of symptoms with the possibility of adverse effects of antidepressants on the foetus and timing of treatment [22, 33].

Factors that influenced prescribing practices included the information available about the safety of antidepressant medication in pregnancy, belief that pregnant depressed women should be treated differently from non-pregnant depressed women, concerns over the legal liability and patient concerns [23]. Female FP’s also acknowledged that their personal experience of pregnancy influenced treatment decisions [33]. FP’s reported using different sources of information on antidepressant use in pregnancy including consultation with pharmacists, formulary issues, relevant manufacturers, the internet and specialist advice [22, 34]. FP’s reported that they felt hesitant to prescribe and tapered dosages of antidepressants rather than discontinuing medication [23]. FP’s relied on their own professional experience and knowledge of individual women to make complex risk-benefit treatment decisions [33]. Professional experience was used to determine the level of involvement that women wanted in the decision-making process [33].

### Available supports

Across the studies FPs reported making referrals to mother–baby units, counsellors, psychiatrists, mental health specialist, local mental health teams, midwives, health visitors, community support groups, voluntary organisations, telephone/crisis line and naturopath [19, 28, 30, 31, 33].

#### Barriers to service provision

Barriers to provision of effective PMH care were identified across studies and are reported here under three subthemes: service user, physician and system level barriers.

### Service user

A reluctance of women to ask for help, denial/non-acceptance of women with current symptoms of perinatal depression and perceived stigma associated with PMHPs were identified as barriers to screening and treatment by FPs [19, 28]. Furthermore, FP’s were reluctant to use the label ‘PPD’ with women because of the stigma that they perceived women felt, the effect this would have on the consultation and because they felt women would recover without formal interventions [31]. However, other FP’s in the same study described consultations where women were happy to accept the label ‘PPD’ [31]. Language barriers or family beliefs were reported by 23% of FP’s as barriers to treatment [28]. While, Edge [32] found that some staff appeared to adopt a ‘colour-blind’ approach to caring for women from diverse ethnic groups instead concentrating on language barriers. It was acknowledged that Black Caribbean women’s psychological responses were interrelated to their cultural identity and that this may affect their comfort in seeking support for mental health issues from HCPs or from social/family resources [32]. Lack of consultation with FP’s by women led to abrupt stopping of antidepressants [33]. FP’s viewed involvement of women in treatment decisions as central to women’s empowerment but this was limited by clinical complexities and the level to which women wanted to be involved in decisions about medications in pregnancy [33].

### Physician

FP’s recognition of their responsibility for PMH care influences professional behaviours and the majority of FPs identified their role in the diagnosis and management of perinatal depression. FPs were more likely to feel responsible for and confident in treating maternal depression than obstetricians and paediatricians [19]. However, a lack of responsibility for follow-up care was identified as a barrier to screening and treatment for PMHPs [19]. Furthermore, Chew- Graham et al. [30] reported that changes to National policy and local organisations influenced care with no one HCP assuming overall responsibility for care of women with PPD. FPs in Brazil [36] saw PPD as the responsibility of psychiatrists in relation to identification, diagnosis and treatment.

Time was reported across the studies as a barrier to screening and treatment of PMHPs [19, 28, 36]. A significant number of respondents in Seehusen et al.’s [27] study believed that screening at every postpartum visit (19.2%) and well-child visit (34.9%) would take too much effort. Similarly, FP’s in McCauley and Casson [33] identified increasing workloads as barriers to complicated treatment decisions.

FP’s reported a wide variety of knowledge and awareness of PMHPs. FP’s had similar awareness scores for perinatal depression compared to both midwives and MCHNs and depression was more likely to be considered postnatally across all three groups [29]. Multifactorial causes of PPD were identified including attributing PPD as a social response to birth and the transition to parenthood [30, 31]. FPs expressed ambivalence about the status of PND as a unique separate condition when compared with depressive illness experienced by women at other times in their lives [30, 31]. A lack of knowledge was reported as a barrier to screening and treatment for PMHPs by some FPs [19]. FP’s in Brazil reported limited knowledge, awareness and recognition and direct clinical experience of caring for women who experience PPD [36]. They viewed PPD as an uncommon problem attributed to hormonal changes [36]. A lack of knowledge was evident around the consequences of perinatal depression with only 20% of FPs in the study by Ververs et al. [22] recognising the negative effects of depression and anxiety on a child’s development. FP’s also reported a lack of awareness of culturally specific issues [32].

Formal training on PPD was received from a variety of sources including residency training, medical literature and through continuing medical education conferences [27]. A lack of confidence, competence and training in identifying and managing PMHPs irrespective of ethnic or cultural backgrounds was reported [23, 32, 36]. More training on mental health issues in the form of continuing professional development opportunities, guidelines specific to PMH and computer deliverables was suggested to support FP’s [19].

### System level barriers

FP’s reported low usage of guidelines in practice due to lack of time and the volume of available guidelines [32, 33]. FPs acknowledged that guidelines provide best practice advice, a professional reference point and can be used as a defence against litigation. However, guidelines were also identified as generic and lacking in specific and clear direction on treatment in the perinatal period. It was also reported that guidelines may be restrictive and may inhibit flexibility and knowledge resulting in women’s individual needs not being met [33]. A lack of specific or accurate guidance was described as a barrier to information provision and led to under treatment of pregnant women in general practice [33, 36].

A lack of available and timely access to resources, the absence of clearly defined care pathways and insufficient specialist PMH services were identified as a barrier to treatment for women with PMH issues [28–33, 36]. In a further study, 29% of GPs reported never referring a woman who is pregnant and on anti-depressants to a psychiatrist and 50% only referred occasionally [22]. In the study by Leiferman et al. [19], 62.8% of FPs reported never/rarely referring patients for treatment for maternal depression. A lack of cultural competence in services acted as a barrier to detection of PMHPs [32]. Inadequate continuity of care, delayed access to treatment and health services offering limited appointment availability were identified as barriers to disclosure and identification of perinatal depression [31, 33].

## Discussion

The aim of this integrative review was to examine the totality of evidence relating to FP’s perceptions and experiences of identifying and caring for women who experience PMHPs. This review identifies a number of aspects to consider within the FP role and service provision in the broader context such as approaches and factors that influence screening, management of PMHPs and barriers to service provision including access to appropriate referral pathways and training opportunities. A summary of the synthesis and recommendations are provided in Table 7.

**Table 7:** Summary of synthesis

Theme	Findings	Limitations of current evidence	Recommendations
1. Identification of PMHPs^a^	A lack of consistent approach to screening for perinatal depression and anxiety evident. Limited use of validated screening tools to aid identification of women experiencing psychological distress.	None of the included studies specifically explored FPs^b^ approach to identifying perinatal psychological distress in primary practice. Studies predominantly examined and explored identification of PPD^c^.	Universal screening for perinatal depression and anxiety using short validated screening tools to be considered for primary care. Explore perinatal mental wellbeing at all antenatal and postnatal interactions with women and their partners. Training opportunities are required to prepare FPs^b^ to incorporate validated screening tools into primary practice. Further research to explore current screening practices including the specific cues and observations that alert FPs^b^ to the possibility of PMHPs^a^.
2. Management of PMHPs^a^ in Primary care	Pharmacological management of PMHPs^a^ was identified as the main treatment modality offered to women in primary care.	The review identified studies which predominantly focused on pharmacological management and made limited reference to non-pharmacological management of PMHPs^a^.	FPs^b^ require support with perinatal pharmacological treatment decisions for women experiencing PMHPs^a^. FPs^b^ require access to a variety of PMH^d^ specific treatment interventions including both pharmacological and non-pharmacological options. Further research is required to identify the non-pharmacological options available to and required by FPs^b^.
Barriers to service provision			
3a. Service user	A reluctance of women particularly from minority ethnic and diverse cultural backgrounds to ask for help because of the perceived stigma associated with PMHPs^a^.	Only one study explored FPs^b^ encounters with Black and minority ethnic women experiencing PMH^d^ care.	National campaigns are required to increase awareness of the spectrum of PMHPs^a^ and encourage women and their families to seek support. Stigma at an individual, public and service level needs to be addressed through awareness and availability of resources and supports. Further research to explore FPs^a^ encounters with women from diverse ethnic and minority groups to identify support mechanisms required by FPs^b^.
3b. Physician level	A lack of knowledge and skills were reported as barriers to screening and treatment of PMHPs by FPs^a^.	Only one study evaluated the training and education needs of FPs^a^ in relation to PMH^d^.	An exploration of FPs^b^ training and education needs in relation to PMH^d^ would ensure that education strategies and professional development opportunities are appropriately contextualised to the needs of FPs^b^.
3c. System level	A lack of available and timely access to resources, clearly defined care pathways and specialist PMH^d^ services.	Included studies did not examine the PMH^d^ referral support needs of FPs^b^.	FPs^b^ require timely access to a range of culturally sensitive and PMH^d^ specific services. A family approach to PMH^d^ care has to be considered to support the woman and family as a whole.

^a^ *PMHPs* perinatal mental health problems

^b^ *FP* Family Physician

^c^ *PPD* Postpartum Depression

^d^ *PMH* perinatal mental health

A low level of identification of women who require support has been cited as a significant barrier to providing more effective PMH care to women and their families [13]. This review identified variable screening practices across studies with clinical discussion identified as the main method of identification similar to findings by Khan [13]. Variations in screening practices may be explained by lack of standardised guidelines. Several validated screening tools are available to aid timely detection of perinatal depression and anxiety [37, 38]. The findings suggest that a multimodal approach to screening is required incorporating education and training, PMH specific guidelines and resources to address FPs confidence, knowledge, attitudes and support FPs to combine clinical judgement with screening tools. Furthermore, calls have been made for enhanced screening for antepartum suicidal ideation because pregnant women are more likely than the general population to experience suicidal ideation [39]. The key to effectiveness of PMH screening programmes is a systematic process of following up all positive screening results with further clinical assessment for depression and anxiety and access to effective interventions, which in return has potential to positively impact on outcomes for women and their families [40]. In addition, health promotion campaigns that target PMH awareness for society are required to create awareness and reduce the stigma associated with PMH.

One of the barriers to identification and care of women identified by FPs was the stigma associated with PMHPs, which FPs perceived inhibited women from disclosing their symptoms. In a systematic review and meta-synthesis of qualitative studies focusing on the experience of care for PMHPs for women in the UK, Megnin-Viggars et al. [14] identified that stigma and fears about losing custody of their baby acted as a barrier to disclosure. An earlier systematic review that explored experiences of motherhood among women with severe mental illness (SMI) [41] found that stigma associated with a psychiatric diagnosis was reinforced by also being a parent and prevented women from discussing PMHPs openly and seeking help. Stigma prevents the establishment of a meaningful therapeutic relationship with HCPs, which is essential for disclosure of need [41]. Furthermore, stigma which may exist at an individual level can be reinforced at a systems level where there is a lack of resources and limited options available to support FP’s and women when PMHPs are disclosed or identified through screening. However, in other studies women described consciously inhibiting disclosure of their feelings to FPs because of personal barriers but also because of FP characteristics such as a perception that FPs were not willing to listen [31]. Women who did feel comfortable disclosing their psychological distress described the importance of having a good relationship with their FP [31].

This review has highlighted a range of contextual factors that may influence professional decision-making. Time was consistently identified as a barrier to providing optimal screening and care of women experiencing PMHPs. The longer the consultation with the woman the more likely that rapport will develop with the HCP, which in turn increases the probability that the woman will feel comfortable opening up about her PMH issues [42]. Women’s experience of FP’s as being too busy or unwilling to listen or dismissive of women’s attempts to communicate their psychological distress has been identified as a barrier to person-centred care [14]. FPs reported language as a barrier to diagnosing PMHPs and Ta Park et al. [43] identified the importance of examining the role of linguistic isolation from the general population as a barrier to seeking help for PMHPs. While, Watt et al. suggest that training must support FPs to recognise and adapt to different cultural expressions of psychological distress [44]. FPs identified the difficulty in women receiving timely initial and follow up appointments in busy practices as a system barrier to women receiving care a view corroborated by women [31]. Negative perceptions of FPs were associated with feeling rushed through consultations or being unable to make appointments due to a lack of FP availability [45].

One of the system barriers identified in this review was a lack of available PMH services and Newman et al. [46] contends that without sufficient resources it is difficult for service providers to offer a variety of effective pathways to recovery. Professional decision-making may be influenced by availability of PMH service referral options and integrated care pathways and there is evidence from this review that where FPs do not have access to these referral pathways that this influences the identification and treatment of women. Milgrom et al. [40] argue that screening without the availability of effective treatment options will be ineffective in reducing morbidity or improving outcomes for women and families. Systematic screening and specific referral pathways that incorporate a range of PMH health services including access to infant mental health interventions in the community are required to support FPs in the identification and treatment of PMHPs [47].

Consistent with the literature, findings of this review were that the primary mode of treatment offered by FPs was pharmacological treatment options. Only a minority of women require pharmacological treatment and the effectiveness of psychological therapies for treatment of perinatal depression and anxiety has been established [48]. Significantly, Megnin-Viggars et al. [14] identified the importance of the FP-service user relationship in the context of treatment decisions where women valued a discussion with the FP that addressed their fears about anti-depressants. While Slade [49] identified the importance of HCP’s interpersonal skills, their ability to form a relationship and engage women who have PMHPs as key determinants of women’s decision to accept help and successful outcomes including linking women with the appropriate interventions.

Counselling was identified as an option provided by FPs in two studies. This option is worthy of note given that both pharmacological and non-pharmacological treatment options need to be available and considered for effective treatment of PMHPs. Findings from a small randomised controlled trial (*n* = 68) suggest that FP management of PPD when augmented by a Cognitive Behavioural Therapy counselling package may be successful in reducing depressive symptoms in women compared to FP management alone [40].

PMH training by FPs was predominantly undertaken in residency training and ongoing education was primarily through reviewing literature rather than formal training opportunities specific to PMH. The importance of training key primary care professionals towards improving current treatment pathways for PPD has been highlighted [40]. There is evidence that training may be effective in increasing FPs effectiveness in identification of PMHPs [13]. Specific PMH training for trainee and qualified FPs is required and consideration should be given to multidisciplinary education programmes, which would enable HCPs with a remit for PMH to dialogue and gain a greater understanding of each HCPs role in PMH care.

### Implications for policy, practice, research and education

Perinatal mental health issues are recognised as an important cause of morbidity and mortality for women, their babies and families and requires healthcare systems across the world to address this area to ensure a consistent approach to screening for PMHPs and equality of access to PMH services and interventions. FPs as the first access point for prevention and early identification of PMH concerns, are ideally placed to meet this agenda but require supports to optimise their role in PMH. FPs require timely access to a range of culturally sensitive PMH services to optimise their ability to support women, their babies and families who experience PMHPs. When FPs have access to PMH specialist health services including a range of psychological and infant mental health interventions this could potentially lead to effective treatment engagement and improve short and long- term outcomes for women, their babies and families. All interactions with women and their families may serve as an opportunity to identify PMHPs and FPs appear open to incorporating a brief validated screening tool into primary practice. Research that examines PMH service needs of FPs has the potential to inform policy development in this area. This review highlights the need for further research to explore the type of screening that is being undertaken by FP’s, specifically screening tools used e.g. clinical interviews, and factors that facilitate effective PMH care in primary care. Furthermore, FPs require guidance on optimising women’s involvement in treatment decisions. Research that examines training needs in relation to PMH and the preferred format of education could be used to inform FP training programmes and curriculum development around PMH (Table 7).

### Strengths and limitations

We employed a robust methodology to identify, select, appraise and synthesise the evidence from a broad range of qualitative and quantitative studies. In addition, we adhered to the relevant standardised reporting guidelines to conduct and report the findings. These methods serve to capture the totality of evidence with respect to the care and management of PMHPs from the perspective of FPs. We also included studies from a broad range of countries, thus enhancing the generalisability of the findings. However, the findings need to be considered in the context of the study limitations. While the research team developed the search string a single reviewer performed the literature search in consultation with a librarian and screened title and abstracts. Only research articles from 2000 onwards have been included and additional studies that would add to the current body of literature may have been excluded from the review. The process of combining papers with different methodologies and frameworks may result in inaccuracies and bias however it is also seen as a strength of the IR methodology as it provides a comprehensive synthesis of varied perspectives from published evidence.

## Conclusion

This IR has highlighted barriers and facilitators that influence FPs’ practice in PMH care and findings are relevant to the current discourse. The collective interpretation revealed that FP’s recognise their role in relation to PMH care however FPs receive variable preparation for this role, there is no consistent approach to screening, the main treatment modality identified was pharmacological management of mood disorders and FPs reported limited access to PMH services which has implications for FPs decisions around pharmacology. Family physicians require access to culturally appropriate services to improve detection and treatment of women from different cultural backgrounds. A biopsychosocial model or approach incorporating education, management and promotion of PMH is required to optimise care to women and their families in the perinatal period.

## Abbreviations

FP: Family Physician
GP: General Practice
HCP: Healthcare Professional
MCHNs: Maternal Child Health Nurses
PMH: Perinatal Mental Health
PMHPs: Perinatal Mental Health Problems
PPD: Postpartum Depression
UK: United Kingdom
US: United States
